# Soluble Angiotensin-Converting Enzyme 2 as a Prognostic Biomarker for Disease Progression in Patients Infected with SARS-CoV-2

**DOI:** 10.3390/diagnostics12040886

**Published:** 2022-04-01

**Authors:** Noelia Díaz-Troyano, Pablo Gabriel-Medina, Stephen Weber, Martin Klammer, Raquel Barquín-DelPino, Laura Castillo-Ribelles, Angels Esteban, Manuel Hernández-González, Roser Ferrer-Costa, Tomas Pumarola, Francisco Rodríguez-Frías

**Affiliations:** 1 Biochemistry Department (Clinical Laboratories), Vall d’Hebron University Hospital, 08035 Barcelona, Spain; noelia.diaz@vhebron.net (N.D.-T.); pgabriel@vhebron.net (P.G.-M.); rbarquin@vhebron.net (R.B.-D.); laura.castillo@vhebron.net (L.C.-R.); aesteban@vhebron.net (A.E.); roferrer@vhebron.net (R.F.-C.); 2 Vall d’Hebron Research Institute, 08035 Barcelona, Spain; manhernandez@vhebron.net (M.H.-G.); tpumarola@vhebron.net (T.P.); 3 Universitat Autònoma de Barcelona, Bellaterra, 08193 Barcelona, Spain; 4 Roche Diagnostics GmbH, 82377 Penzberg, Germany; stephen.weber@roche.com (S.W.); martin.klammer@roche.com (M.K.); 5 Immunology Department (Clinical Laboratories), Vall d’Hebron University Hospital, 08035 Barcelona, Spain; 6 Microbiology Department (Clinical Laboratories), Vall d’Hebron University Hospital, 08035 Barcelona, Spain

**Keywords:** disease severity, COVID-19, SARS-CoV-2, angiotensin-converting enzyme 2, SARS-CoV-2 spike protein, biomarkers, inflammation

## Abstract

Predicting disease severity in patients infected with SARS-CoV-2 is difficult. Soluble angiotensin-converting enzyme 2 (sACE2) arises from the shedding of membrane ACE2 (mACE2), which is a receptor for SARS-CoV-2 spike protein. We evaluated the predictive value of sACE2 compared with known biomarkers of inflammation and tissue damage (CRP, GDF-15, IL-6, and sFlt-1) in 850 patients with and without SARS-CoV-2 with different clinical outcomes. For univariate analyses, median differences between biomarker levels were calculated for the following patient groups (classified by clinical outcome): RT-PCR-confirmed SARS-CoV-2 positive (Groups 1–4); RT-PCR-confirmed SARS-CoV-2 negative following previous SARS-CoV-2 infection (Groups 5 and 6); and ‘SARS-CoV-2 unexposed’ patients (Group 7). Median levels of CRP, GDF-15, IL-6, and sFlt-1 were significantly higher in hospitalized patients with SARS-CoV-2 compared with discharged patients (all *p* < 0.001), whereas levels of sACE2 were significantly lower (*p* < 0.001). ROC curve analysis of sACE2 provided cut-offs for predicting hospital admission (≤0.05 ng/mL (positive predictive value: 89.1%) and ≥0.42 ng/mL (negative predictive value: 84.0%)). These findings support further investigation of sACE2, as a single biomarker or as part of a panel, to predict hospitalization risk and disease severity in patients with SARS-CoV-2 infection.

## 1. Introduction

Severe acute respiratory syndrome coronavirus 2 (SARS-CoV-2) is the causal agent of coronavirus disease 2019 (COVID-19), the global outbreak of which was declared a pandemic on 11 March 2020 by the World Health Organization (WHO) [1]. The virus predominantly targets the respiratory system, initially causing local virus-mediated tissue damage, followed by a second phase in which infected host cells trigger an immune response. In severe SARS-CoV-2 infection, this activation of the immune system results in a cytokine storm, causing a local and systemic inflammatory response, leading to dysfunction and damage in other organ systems [2]. Any proposed biomarker algorithm for SARS-CoV-2 disease progression should therefore include markers of inflammation and tissue damage. Presently, there are no established prognostic models for predicting SARS-CoV-2 disease progression; a recent systematic review by Wynants et al. critically appraised 107 such prognostic models; however, only one was proposed to be scientifically robust and warranting further clinical investigation [3].

There are many biomarkers of inflammation and tissue damage associated with SARS-CoV-2 prognosis [4]. Biomarkers of immune system activation, such as pro-inflammatory cytokine mediators (including interleukin-6 (IL-6)) and proteins of heterogeneous functions that can be activated in pro-inflammatory states (including C-reactive protein (CRP), growth/differentiation factor-15 (GDF-15), and soluble fms-like tyrosine kinase-1 (sFlt-1)), may play a role in SARS-CoV-2 pathology. IL-6 and CRP are known to be associated with inflammatory processes in severe SARS-CoV-2 infection [5,6,7,8,9,10], whereas GDF-15 and sFlt-1 are associated with SARS-CoV-2-mediated tissue damage [11,12,13].

Membrane-bound angiotensin-converting enzyme 2 (mACE2) serves as a receptor for the spike protein of SARS-CoV-2 [14] and is also a part of the renin–angiotensin system (RAS). In cardiovascular disease, the classic RAS arm is commonly dysregulated and exerts most of its pathological effects through the peptide hormone angiotensin II. In contrast, ACE2 and its products angiotensin-(1–7) and angiotensin-(1–9) form the counter-regulatory arm of the RAS system and have protective effects at the pulmonary (antifibrotic) and cardiovascular (vasodilator) levels [15,16,17]. Pathological changes that occur in patients with severe SARS-CoV-2 infection—such as increased vascular permeability, local tissue injury, and fibrosis—have been attributed to a reduction in mACE2 activity due to binding of SARS-CoV-2 [18,19].

Angiotensin II stimulates the shedding of soluble ACE2 (sACE2) from mACE2 through a disintegrin and metalloproteinase-17 (ADAM-17) [20]. The risk/benefit profile of sACE2 in the context of SARS-CoV-2 infection is disputed. Swärd et al. (2020) proposed that elevated sACE2 levels reflect high mACE2 and/or increased shedding of mACE2 via ADAM-17 and could therefore have value as an indicator of risk for severe SARS-CoV-2 infection [21]. Meanwhile, Leow (2020) describes the potential benefits of elevated sACE2 levels, whereby sACE2 could function as a decoy ligand and maintain its affinity for SARS-CoV-2 through the spike protein while being unable to mediate cellular entry of the virus [6].

There is an unmet clinical need for better prediction of disease severity in patients with SARS-CoV-2 infection to inform patient management and ensure timely treatment. While sACE2 is known to be involved in the pathophysiology of COVID-19, it is yet to be thoroughly investigated as a biomarker of disease severity. Given that sACE2 is a novel biomarker under evaluation, we evaluated its prognostic value in the context of other known biomarkers of inflammation and tissue damage (CRP, GDF-15, IL-6, and sFlt-1) in patients with and without SARS-CoV-2 infection who experienced different clinical outcomes.

## 2. Materials and Methods

### 2.1. Study Design and Ethical Statement

This was a prospective, observational, single-center study conducted at Vall d’Hebron University Hospital (Barcelona, Spain) between March and October 2020. The study complied with the principles of the Declaration of Helsinki and received approval (reference: PR [AG] 577/2020) on 10 November 2020 from the Research Ethics Committee for Drug Research of the Vall d’Hebron University Hospital (Barcelona, Spain). Patient-level data were processed in accordance with Regulation (EU) 2016/679 of the European Parliament on Data Protection. An exemption from obtaining patient informed consent was granted by the Research Ethics Committee due to the health emergency presented by the COVID-19 pandemic.

### 2.2. Patients

Adults aged 25–90 years presenting to the emergency department and primary care clinic of Vall d’Hebron University Hospital were sampled by nasopharyngeal swab for detection of SARS-CoV-2 infection by reverse transcription polymerase chain reaction (RT-PCR) testing (cobas^®^ SARS-CoV-2 real-time RT-PCR test; Roche Diagnostics International Ltd., Rotkreuz, Switzerland). Patients with cancer or pathologies related to autoimmune or cardiovascular diseases were excluded, as well as patients who were being treated with immunosuppressant therapy. The following comorbidities were included for the full cohort analyses: hypertension, type 2 diabetes mellitus, and dyslipidemia. Additional analyses were conducted on a sub-cohort excluding these comorbidities.

Serum samples were collected from all patients admitted to hospital from March to October 2020, for whom routine clinical assessment of IL-6 and CRP was requested. Samples were frozen at −80 °C in a Biobank repository until analysis. All patients underwent RT-PCR analysis for SARS-CoV-2 infection at the time of blood draw (with the investigator being blinded to the result of the analysis). Samples were selected at random from the repository and the SARS-CoV-2 infection status of the patient and their clinical outcomes were revealed after selection. IL-6 and CRP measurements of the patient were retrieved from a database for further analysis. Recruited patients with RT-PCR-confirmed positive SARS-CoV-2 infection at the time of blood draw were grouped according to clinical outcome (Table 1): emergency care and home discharge (Group 1); ward admission for moderate illness (Group 2); admission to the intensive care unit (ICU; Group 3); and death associated with SARS-CoV-2 infection (Group 4). Patients who tested negative for SARS-CoV-2 following a previous infection were divided into two groups: an independent cohort of patients who tested negative for SARS-CoV-2 infection 1 month after finishing a 15-day quarantine following previous RT-PCR-confirmed SARS-CoV-2 infection that required emergency care and home discharge (Group 5), and a cohort of patients from Group 3 previously admitted to the ICU, who were tested for SARS-CoV-2 infection immediately before discharge from the hospital and were negative (Group 6). These patients were included to examine how the biomarkers behaved after a mild infection and whether the biomarkers recovered to normal levels, or if changes in biomarkers were only observed after a severe infection. A subset of patients had two samples taken and tested with RT-PCR: an initial sample that was confirmed positive for SARS-CoV-2 (Group 3); and a second sample that was taken when these patients were ready to be discharged which was negative (Group 6). Data were not available regarding how long a patient had been symptomatic of SARS-CoV-2 infection before attending the hospital or the disease severity at the time of blood sampling; however, samples were drawn prior to any of the above-mentioned clinical outcomes. SARS-CoV-2 negative patient samples (‘SARS-CoV-2 unexposed’ patients) were from adults aged 25–90 years who tested negative for SARS-CoV-2 infection following RT-PCR of a nasopharyngeal swab or had no medical history of SARS-CoV-2 infection (Group 7). These patients were included to better understand the behavior of the biomarkers in an ‘unexposed’ population. Treating physicians had access to CRP and IL-6 values but GDF-15, sACE2, and sFlt-1 results were not available.

### 2.3. Sample Handling

Anonymized serum samples were thawed and the aliquots were stored at 2–8 °C during the week of testing. Following testing, all samples were stored in a serum bank at −80 °C until measurements for all biomarkers were completed. For the SARS-CoV-2 negative patient group, serum samples were stored in the serum bank at −80 °C for consistency with the RT-PCR-confirmed SARS-CoV-2 positive serum samples.

### 2.4. Assays

Serum GDF-15, IL-6, and sFlt-1 levels were measured using the Elecsys^®^ GDF-15, Elecsys IL-6, and Elecsys sFlt-1 electrochemiluminescence immunoassays, respectively, according to the manufacturer’s instructions on the Cobas 8000 analyzer (all Roche Diagnostics International Ltd. (Rotkreuz, Switzerland)). IL-6 was only measured in samples where it had not been previously assessed during routine analysis. Serum CRP levels were measured using an immunoturbidimetry assay on the AU5800 analyzer (Beckman Coulter, Brea, CA, USA). Serum sACE2 levels were measured using a human ACE2 sandwich enzyme-linked immunosorbent assay (ELISA; RayBiotech, Atlanta, GA, USA) on the Grifols Triturus analyzer (Grifols Diagnostic Solutions Inc., Emeryville, CA, USA). Reagents, calibrators, and control materials from the same manufacturer were used for the purpose of this study.

### 2.5. Data Analysis

Sample size was informally calculated according to the estimated number of patients attending the hospital, the service level of the laboratory, and reagent and test availability. Clinical variables for all patients, including demographic data, medical history (including history of liver disease), metabolic profile, and medications were assessed via a review of medical records. Data on the following biochemical variables were also collected: full blood count (including platelets), coagulation (prothrombin time and D-dimer tests), liver function tests (aspartate aminotransferase, alanine aminotransferase, alkaline phosphatase, gamma-glutamyl transferase, and bilirubin), lipids (cholesterol and triglycerides), substrates (glucose), renal function (creatinine and urea), and inflammatory markers (IL-6 and CRP). Any missing data were not further considered for statistical analysis.

All values for the statistical analysis were collected, processed, and analyzed by researchers at the Vall d’Hebron University Hospital. The data were stored in a pre-specified, anonymized, protected database in electronic format (Excel) and kept at the hospital, where researchers performed the data analysis using R statistical software (version 3.6.2).

The statistical significance of the differences in demographic and biochemical variables was assessed using chi-squared test (male sex, chronic kidney disease, blood pressure, type 2 diabetes mellitus, dyslipidemia, and body mass index), one-way analysis of variance (ANOVA; age and mean arterial pressure), and Kruskal–Wallis comparison (aspartate aminotransferase, alanine aminotransferase, prothrombin time, and D-dimer). For univariate biomarker analyses, median differences between biomarker levels in patients with SARS-CoV-2 positive and SARS-CoV-2 negative patients were calculated, and the significance was determined using the Mann–Whitney U test. Statistical significance was assigned where the *p*-value was <0.05. Sensitivity and specificity with 95% confidence intervals (CIs) were calculated, and receiver operating characteristic (ROC) analysis used to evaluate area under the curve (AUC); optimal cut-offs were determined by means of the Youden index. For bivariate biomarker analysis, logistic regression in combination with the mlr R-package [22] was performed. All possible two-biomarker combinations were assessed by means of 10-fold cross-validation; bivariate combinations showing an AUC improvement of ≥1 percentage point over the best univariate biomarker were reported.

To calculate statistical significance (*p*-values) for a biomarker’s AUC difference between the full cohort and the sub-cohort excluding co-morbidities, a bootstrapping procedure was applied. In brief, $X_{L}$ is considered to be the vector of values with length n that corresponds to the biomarker with the larger AUC ($AUC_{L}$) and $X_{S}$ with length m corresponds to the biomarker with the smaller AUC ($AUC_{S}$). Then, i=10,000 random bootstrap samples (i.e., sampling with replacement) $X_{L_{i}}^{*}$ and $X_{S_{i}}^{*}$ with length n and m, respectively, were drawn and their AUC differences *d_i_* (defined as $AUC_{L_{i}}^{*}-AUC_{S_{i}}^{*}$) were calculated. The *p*-value was then computed as $\frac{\left(\#d<0\right)+1}{i}*2$ i.e., the fraction of differences smaller than zero, increased by one to account for the sampling unsharpness, then multiplied by two to account for the two-sided *p*-value.

## 3. Results

### 3.1. Patients

A total of 963 samples from 850 patients were included in the present analyses. Selected demographic, clinical, and biochemical characteristics of each group are shown in Table 2. Across all comparisons between clinical outcomes, the investigated biomarkers did not show a statistically significant difference in their performances between the full cohort and the sub-cohort excluding comorbidities (hypertension, type 2 diabetes mellitus, and dyslipidemia), except for GDF-15 that exhibited a significantly higher AUC in the sub-cohort excluding comorbidities.

### 3.2. Univariate Analysis of Patients Infected with SARS-CoV-2 versus ‘SARS-CoV-2 Unexposed’ Patients

Levels of CRP, GDF-15, IL-6, and sFlt-1 were significantly higher (all *p* < 0.001) in patients infected with SARS-CoV-2 (Groups 1–4) compared with ‘SARS-CoV-2 unexposed’ patients (Group 7), whereas levels of sACE2 were significantly lower (*p* < 0.001) in patients infected with SARS-CoV-2 compared with ‘SARS-CoV-2 unexposed’ patients (Table 3). Based on the analysis of ROC curves, the AUC value was highest for CRP (0.964 [95% CI 0.948, 0.980]), followed by IL-6 (0.949 [0.933, 0.964]), GDF-15 (0.830 [0.797, 0.863]), sFlt-1 (0.797 [0.764, 0.829]), and sACE2 (0.585 [0.539, 0.632]) (Table 3). In univariate analyses of biomarker performance in the full cohort versus the sub-cohort excluding co-morbidities, only GDF-15 was significantly different (AUC: 0.894 [0.856, 0.933], *p*-value: 0.008).

### 3.3. Univariate and Bivariate Analysis of Patients Infected with SARS-CoV-2 Who Were Admitted to Hospital versus Patients Infected with SARS-CoV-2 Who Were Discharged

**Figure 1:**
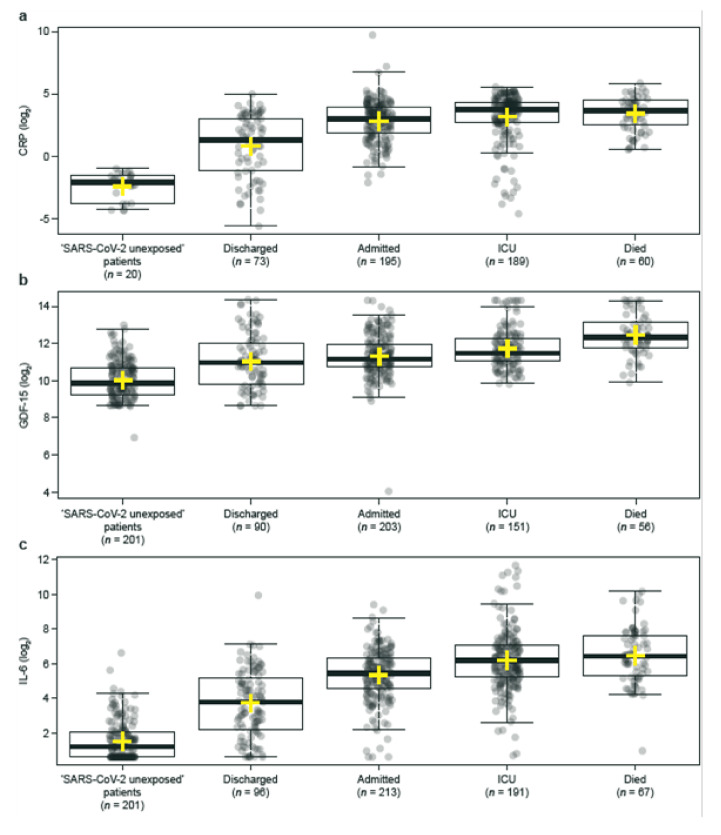

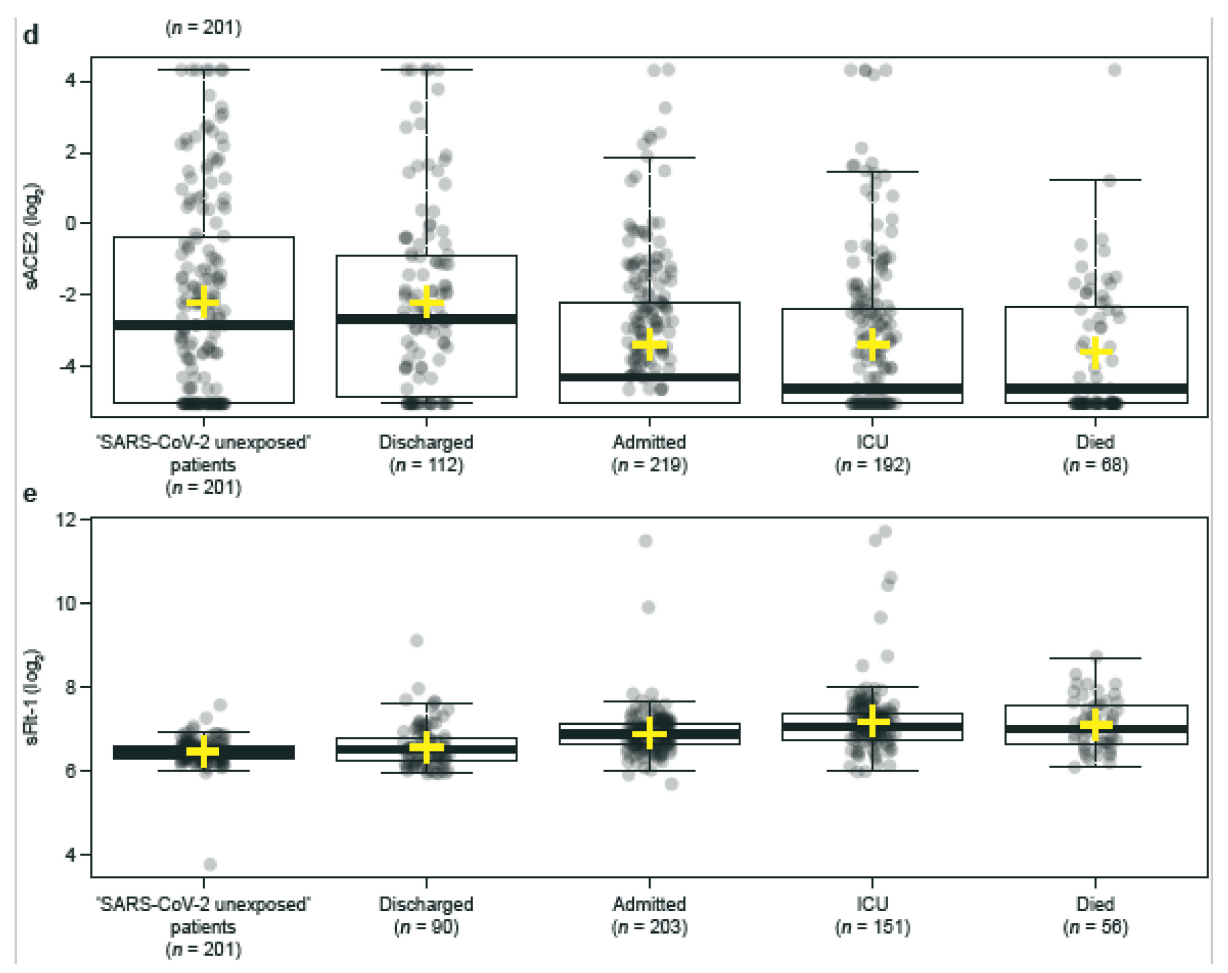
Levels of (**a**) CRP, (**b**) GDF-15, (**c**) IL-6, (**d**) sACE2, and (**e**) sFlt-1 by disease severity in patients with SARS-CoV-2 who were admitted to hospital (Groups 2–4) and patients with SARS-CoV-2 who were discharged (Group 1). Thick black line = median; yellow cross = mean; upper/lower limits and whiskers = interquartile range and maximum/minimum values excluding outliers.

Levels of CRP, GDF-15, IL-6, and sFlt-1 were significantly higher (all *p* < 0.001) in patients infected with SARS-CoV-2 who were admitted to hospital (Groups 2–4) compared with patients infected with SARS-CoV-2 who were discharged (Group 1), whereas levels of sACE2 were significantly lower (*p* < 0.001) in patients infected with SARS-CoV-2 who were admitted to hospital compared with those who were discharged (Table 4). Based on the analysis of ROC curves, the AUC value was highest for IL-6 (0.800 [95% CI 0.750, 0.851]), followed by CRP (0.775 [0.718, 0.832]), sFlt-1 (0.751 [0.689, 0.813]), sACE2 (0.648 [0.592, 0.704]), and GDF-15 (0.625 [0.551, 0.699]) (Table 4). Furthermore, median levels of CRP, GDF-15, IL-6, and sFlt-1 increased with COVID-19 disease severity, whereas the median level of sACE2 decreased with COVID-19 disease severity (Figure 1). In univariate analyses of biomarker performance in the full cohort versus the sub-cohort excluding co-morbidities, only GDF-15 was significantly different (AUC: 0.795 [0.696, 0.894], *p*-value: 0.012).

**Figure 2:**
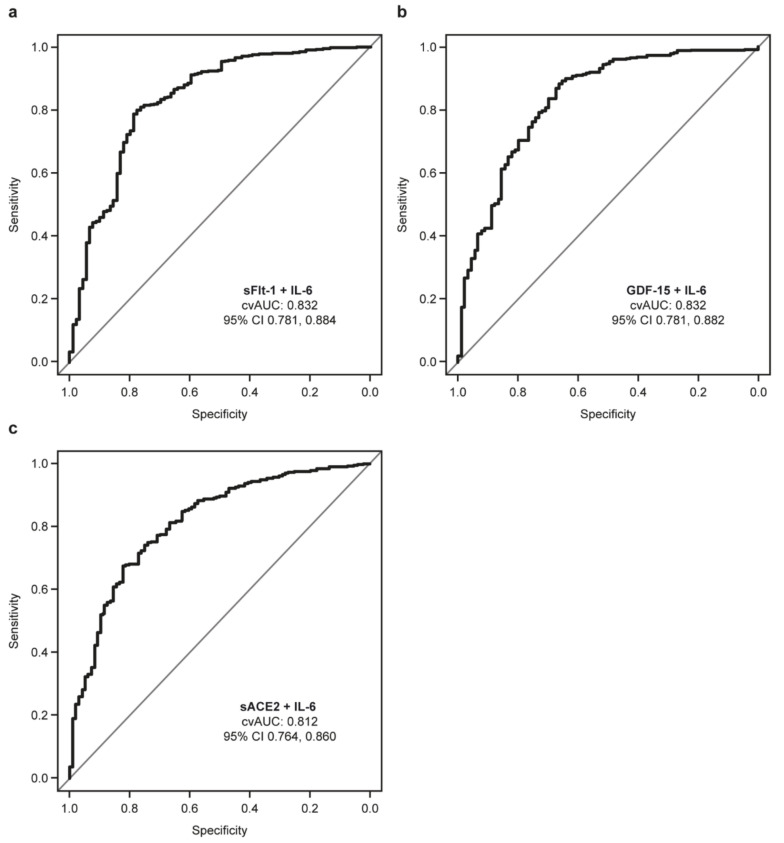
Bivariate analysis performed in patients with SARS-CoV-2 infection who were admitted to hospital (Groups 2–4) versus patients with SARS-CoV-2 infection who were discharged (Group 1). Data are shown as ROC curves for (**a**) sFlt-1 + IL-6, (**b**) GDF-15 + IL-6, and (**c**) sACE2 + IL-6.

Bivariate analysis based on ROC curves (Figure 2) showed that the addition of sFlt-1 (cross-validated (cv) AUC: 0.832), GDF-15 (cvAUC: 0.832), or sACE2 (cvAUC: 0.812) to IL-6 provided an improvement in AUC value compared with IL-6 alone (AUC: 0.800) in patients with SARS-CoV-2 who were admitted to hospital (Groups 2–4) versus patients with SARS-CoV-2 who were discharged (Group 1).

**Figure 3:**
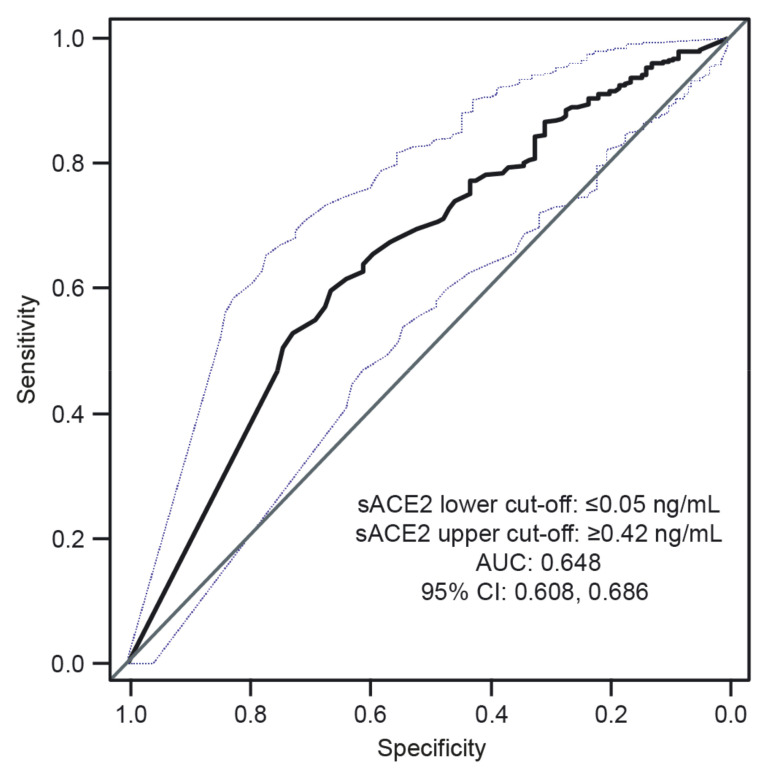
ROC curve analysis used to calculate lower and upper cut-off values of sACE2 for the prediction of hospitalization in patients with SARS-CoV-2. Blue dotted lines = upper and lower limits of the 95% CI for AUC.

As sACE2 has not previously been explored as a biomarker for COVID-19 disease severity, two cut-offs were proposed for sACE2 based on ROC curve analysis for the prediction of hospitalization versus discharge in patients infected with SARS-CoV-2 (Figure 3). A cut-off of ≤0.05 ng/mL produced a positive predictive value (PPV) of 89.1% (95% CI 84.9, 92.5) and a cut-off of ≥0.42 ng/mL provided a negative predictive value (NPV) of 84.0% (95% CI 80.4, 87.1).

### 3.4. Univariate Analysis of Patients Infected with SARS-CoV-2 Who Were Admitted to the ICU or Died versus Patients Infected with SARS-CoV-2 Who Were Admitted to the Ward or Discharged

Levels of CRP, GDF-15, IL-6, and sFlt-1 were significantly higher (all *p* < 0.001) in patients infected with SARS-CoV-2 who were admitted to the ICU or died (Groups 3 and 4) compared with patients infected with SARS-CoV-2 who were admitted to the ward or discharged (Groups 1 and 2). sACE2 levels were significantly lower (*p* = 0.015) in patients who were admitted to the ICU or died compared with patients who were admitted to the ward or discharged. Based on the analysis of ROC curves, the AUC value was highest for IL-6 (0.715 [95% CI 0.673, 0.757]), followed by sFlt-1 (0.672 [0.624, 0.720]), CRP (0.670 [0.623, 0.716]), GDF-15 (0.650 [0.602, 0.698]), and sACE2 (0.556 [0.511, 0.600]) (Table 5). In univariate analyses of biomarker performance in the full cohort versus the sub-cohort excluding co-morbidities, only GDF-15 was significantly different (AUC: 0.748 [0.680, 0.816], *p*-value: 0.022).

### 3.5. Patients Who Were RT-PCR-Confirmed Negative for SARS-CoV-2 Infection Following a Previous Positive RT-PCR Result

Median sACE2 levels were similar (*p* = 0.273) between samples from patients who were RT-PCR-confirmed positive for SARS-CoV-2 at the time of blood draw and received emergency care then discharged at the time of infection (Group 1; 0.160 ng/mL) and samples from a different group of patients who received similar care who were RT-PCR-confirmed negative for SARS-CoV-2 infection at the time of blood draw (Group 5; 0.235 ng/mL). In contrast, median sACE2 levels were significantly higher (*p* = 0.003) in samples from patients who were RT-PCR-confirmed negative for SARS-CoV-2 infection following a previous positive RT-PCR result and admitted to the ICU (Group 6; 0.130 ng/mL), as compared with samples taken from the same patients when they were RT-PCR-confirmed positive for SARS-CoV-2 (Group 3; 0.040 ng/mL).

In all patients who were RT-PCR-confirmed negative for SARS-CoV-2 infection following a positive RT-PCR result (Groups 5 and 6), a significant increase (*p* = 0.049) in sACE2 levels was observed in those who were discharged (median sACE2 level: 0.235 ng/mL) compared with those who were admitted to the ICU (median sACE2 level: 0.130 ng/mL).

## 4. Discussion

We evaluated the value of sACE2 as a novel biomarker for COVID-19 disease severity in the context of other markers of inflammation and tissue damage (CRP, GDF-15, IL-6, and sFlt-1) in a large cohort of patients with and without SARS-CoV-2 infection who experienced different clinical outcomes. Univariate analysis showed that median levels of sACE2 were significantly lower in patients infected with SARS-CoV-2 compared with ‘SARS-CoV-2 unexposed’ patients, as well as in patients infected with SARS-CoV-2 who were admitted to hospital, compared with patients infected with SARS-CoV-2 who were discharged. The same observation emerged in patients infected with SARS-CoV-2 who were admitted to the ICU or who died, compared with patients who were discharged or admitted to the ward. These findings suggest that sACE2 has value as a biomarker for hospitalization and disease severity in patients with SARS-CoV-2 infection. Furthermore, two cut-offs for sACE2 for predicting severe SARS-CoV-2 infection were derived: ≤0.05 ng/mL with a PPV of 89.1% and ≥0.42 ng/mL with a NPV of 84.0%. After future validation in larger studies in an acute care setting, the cut-off of ≤0.05 ng/mL could be used to indicate risk of severe disease when IL-6 or CRP values are not elevated to sufficient levels due to very recent SARS-CoV-2 infection. Meanwhile, a cut-off of ≥0.42 ng/mL could help identify most patients who develop mild disease.

CRP had the highest AUC value when comparing patients infected with SARS-CoV-2 versus ‘SARS-CoV-2 unexposed’ patients, and IL-6 had the highest AUC when comparing patients infected with SARS-CoV-2 who were admitted to hospital versus those who were discharged and when comparing patients infected with SARS-CoV-2 who were admitted to the ICU or died versus those who were admitted to the ward or discharged. Overall, median levels of CRP, GDF-15, IL-6, and sFlt-1 were significantly higher in patients with SARS-CoV-2 infection who had the most severe clinical outcome in all comparisons. It should be noted that all of the present analyses were bivariate with no adjustments made for potential confounders; however, these findings are consistent with previously published studies that have reported the value of these biomarkers for disease severity in patients infected with SARS-CoV-2 infection [9,11,12,23]. Nevertheless, it would be interesting to examine the potential value that the biomarkers studied herein could add to an initial estimate of severe disease risk based purely on demographic and clinical data.

Across all comparisons between clinical outcomes, only GDF-15 exhibited a significantly higher AUC in the sub-cohort excluding comorbidities as compared to the full cohort. This could indicate the relationship between SARS-CoV-2 and the cardiovascular damage caused by infection with the virus. In addition, the other biomarkers measured, including sACE2, appear to be independent of the co-morbidities present in this study population (hypertension, type 2 diabetes mellitus, and dyslipidemia). Across all patients with SARS-CoV-2 infection, sACE2 levels were significantly lower than in SARS-CoV-2 negative patients, which could indicate a reduced amount of mACE2 susceptible to cleavage by ADAM-17. In addition, the median level of sACE2 was significantly lower in patients infected with SARS-CoV-2 with the most severe clinical outcome in all comparisons. These findings support the hypothesis that sACE2 could play a protective role in patients infected with SARS-CoV-2 [6]. One mechanism through which sACE2 could have this protective effect is by remaining residually active and performing negative feedback in the activation of RAS. In keeping with this, human recombinant sACE2 has been explored as a treatment option for patients infected with SARS-CoV-2 and was associated with a decrease in concentration of critical cytokines implicated in COVID-19 pathology, reductions in viral load, and neutralization of viral particles; however, speculation remains whether these observations were reflective of the sACE2 treatment or the natural course of the viral infection [24,25].

In contrast to our findings, a recent study by Kragstrup et al. reported that high plasma ACE2 was associated with increased maximal illness severity within 28 days; however, the patient population in the study by Kragstrup et al. was different to the population in the current study in that the study by Kragstrup et al. did not examine longitudinal samples from the same patient during hospitalization. In addition, plasma ACE2 was measured using a different methodology (protein extension assay), which does not allow the determination of a cut-off value for prediction of severe disease and limits the comparability of findings with other studies [26]. Another study in a small number of patients reported increased sACE2 in patients with severe COVID-19 [27]; however, this increase has been hypothesized to be a transient response due to increased shedding from infected cells [28]. Thus, the timing of sampling from a positive SARS-CoV-2 RT-PCR test may be an important factor in interpreting sACE2 levels. Local sACE2 levels may be a more accurate indicator of COVID-19 severity and explain the differences in response to infection, degree of disease severity, and recovery period observed between patients. Circulating levels of sACE2 have also been shown to vary by sex and age [21,29]. A recent study by Wang et al. showed that sACE2, alongside other RAS pathway molecules such as angiotensin-(1–7) and membrane-bound vs. soluble tumor necrosis factor receptors I and II as a surrogate marker of ADAM-17 activity, were dysregulated in patients with SARS-CoV-2 infection. This suggests that there is a crucial role for ADAM-17 activation and ACE2 shedding in SARS-CoV-2 pathophysiology [29].

In patients infected with SARS-CoV-2 who were admitted to the ICU, levels of sACE2 were significantly higher in blood samples drawn after the resolution of the infection (0.130 ng/mL) compared with blood samples drawn at the time of RT-PCR-confirmed positive for SARS-CoV-2 (0.040 ng/mL). These data suggest that levels of sACE2 can rise after infection. In further support of a protective role of sACE2, samples taken from patients infected with SARS-CoV-2 who were admitted to the ICU had approximately half the sACE2 levels of those samples taken after resolution of viral infection (0.130 vs. 0.235 ng/mL).

One strength of this study is the large number of samples from different patient groups that were used to determine median biomarker levels. In addition, the observed age range of all individuals included in this study (48.2–71.6 years) was reflective of the group most likely to be at risk of more severe clinical outcomes when infected with SARS-CoV-2, relative to younger patients [30].

Limitations of this study include the single-center design and under-representation of certain groups (i.e., patients infected with SARS-CoV-2 who were discharged and SARS-CoV-2 negative patients). In addition, the patients recruited with mild symptoms of SARS-CoV-2 infection were relatively complicated in terms of their clinical presentation, as they were recruited from the emergency department. Given the public health recommendation to stay at home during the period of this study, it was not possible to obtain samples from patients with mild symptoms who did not require hospital treatment and both the severity and time of symptom onset were highly variable among patients. The timing of sampling from a positive SARS-CoV-2 RT-PCR test was also variable. Furthermore, it is important to note that sACE2 is expressed in other organs in addition to the lungs [31], meaning that a blood draw is indicative of universal circulating levels of sACE2, not just sACE2 expressed by the lungs. Medication use was not recorded; therefore, the effect of concomitant medication use (e.g., ACE inhibitors or angiotensin receptor blockers, statins, and metformin, respectively) on the biomarker measurements was unknown.

sACE2 measurements in this study were performed using a non-commercial kit for which the quality specifications are not as robust as those of the other markers used in clinical practice. Moreover, the sACE2 cut-offs derived for prediction of mild and severe SARS-CoV-2 infection in the present study may not be generalizable to other institutions utilizing different sACE2 assays. Furthermore, any biomarker assay that would be useful for triage in an emergency department or primary care clinic would need to be fully automated with a turnaround time that did not hamper its clinical utility.

Future validation of the present biomarker measurements in an independent cohort of patients is warranted. In particular, the effect of COVID-19 vaccination status and novel SARS-CoV-2 strains should be considered. Longitudinal studies of sACE2 levels in patients who have recovered from severe SARS-CoV-2 infection are required to confirm whether sACE2 levels increase after a total recovery of symptoms or if they are permanently reduced following severe SARS-CoV-2 infection. Furthermore, long-term studies of sACE2 levels are required in at-risk populations (e.g., those with and without hypertension and chronic kidney disease) and in patients receiving treatments that may affect sACE2 activity or concentration (i.e., ACE inhibitors or angiotensin receptor blockers). In addition, evaluation of other inflammatory markers associated with SARS-CoV-2 disease severity—such as D-dimer, ferritin, neutrophil-to-lymphocyte ratio, platelet-to-lymphocyte ratio, and comparison with sACE2 for prediction of SARS-CoV-2 prognosis—could aid in understanding the mechanisms behind the present findings [32,33]. To promote reproducibility between studies, future works should more closely align clinical outcomes with standardized measures of clinical outcomes (e.g., the WHO clinical progression scale for COVID-19) [34].

In conclusion, while the present findings have shown limited clinical utility of sACE2 as a novel biomarker for hospitalization risk and disease severity in patients infected with SARS-CoV-2, the results provide a basis for future investigations into sACE2 in independent populations or in longer-term studies.

## Figures and Tables

**Table 1:** Summary of sample cohorts.

Group	Sample Cohort	Number of Samples	Sample Cohort Characteristics (Symptoms, Clinical Evaluation, and Management)
1	Emergency care and home discharge *	112	Patients who presented to the ED with symptoms suggestive of SARS-CoV-2 infection, which was confirmed by SARS-CoV-2 positive RT-PCR, but who did not require hospital admission as they had mild symptoms or were asymptomatic. In general, these patients underwent home isolation and some required oxygen support in the ED.
2	Ward admission for moderate illness	219	Patients who presented to the ED with symptoms suggestive of SARS-CoV-2 infection, which was confirmed by SARS-CoV-2 positive RT-PCR, and who were subsequently hospitalized. These patients had radiological findings compatible with SARS-CoV-2 pneumonia and required non-invasive ventilator support (e.g., Ventimask, nasal cannulas).
3	Admission to the ICU	192	Hospitalized patients who were admitted to the ICU due to worsening of SARS-CoV-2 pneumonia. Most of these patients required invasive ventilator support in the form of orotracheal intubation and oxygenation through an extracorporeal membrane.
4	Death associated with SARS-CoV-2	68	Hospitalized patients who died due to SARS-CoV-2 infection. All patients included in this group had a death certificate that listed SARS-CoV-2 pneumonia as the primary cause of death. All of these patients required ventilator support; in some cases, non-invasive ventilator support was provided due to withdrawal of care.
1–4	RT-PCR-confirmed positive for SARS-CoV-2 infection at time of blood draw	591	
5	Emergency care and home discharge	58	Patients who presented to the ED with symptoms suggestive of SARS-CoV-2 infection, which was confirmed by SARS-CoV-2 positive RT-PCR, but who did not require hospital admission as they had mild symptoms or were asymptomatic. In general, these patients underwent home isolation. Blood draws were performed 1 month after the patient finished a 15-day quarantine, or 1 month after the patient no longer displayed symptoms of SARS-CoV-2 infection.
6	Admission to the ICU	113	Hospitalized patients from Group 3 who were admitted to the ICU due to worsening of SARS-CoV-2 pneumonia. Most of these patients required invasive ventilator support in the form of orotracheal intubation and oxygenation through an extracorporeal membrane. Patients in this group were considered SARS-CoV-2 convalescent. Blood draws were performed at the end of the patient’s hospital admission after they were RT-PCR-confirmed negative for SARS-CoV-2 infection.
5–6	RT-PCR-confirmed negative for SARS-CoV-2 infection at time of blood draw after previous RT-PCR-confirmed positive result	171	
7	‘SARS-CoV-2 unexposed’ patients	201	Patients who presented to a primary care setting for routine checks of their chronic pathology or health basic study. All SARS-CoV-2 negative patients were RT-PCR-confirmed negative for SARS-CoV-2 infection or had no medical history of SARS-CoV-2 infection.
Total		963	

* Patients who were admitted for other illnesses and then found to be infected by nosocomial SARS-CoV-2 but were asymptomatic were included in this group (*n* = 18). ED: emergency department; ICU: intensive care unit; RT-PCR: reverse transcription polymerase chain reaction; SARS-CoV-2: severe acute respiratory syndrome coronavirus 2.

**Table 2:** Selected demographic, clinical, and biochemical characteristics of the patient groups.

Variable	Group							*p*-Value *
	RT-PCR-Confirmed Positive for SARS-CoV-2				RT-PCR-Confirmed Negative for SARS-CoV-2			
	1 Emergency Care and Discharge	2 Ward Admission	3 ICU Admission	4 Death	5 Emergency Care and Discharge	6 ICU Admission	7 ‘SARS-CoV-2 Unexposed’ Patients	
Age (years), mean (SD)	59.1 (20.9)	60.7 (15.4)	55.4 (11.7)	71.6 (11.1)	48.2 (17.3)	55.2 (11.8)	61.4 (15.8)	<0.001
Male, n (%)	50 (44.6)	114 (52.1)	116 (60.4)	35 (51.5)	28 (48.3)	69 (61.1)	87 (43.3)	0.006
Chronic kidney disease, n (%) ^†^	23 (20.5)	34 (15.6)	25 (13.1)	22 (32.4)	2 (3.6)	12 (10.6)	10 (5.0)	<0.001
Arterial pressure (mmHg), mean (SD)	96.4 (14.2)	93.7 (12.9)	91.7 (13.4)	90.3 (14.9)	84.6 (11.6)	92.7 (12.0)	ND	0.426
Blood pressure >140/90 mmHg, n (%)	53 (47.3)	115 (52.5)	73 (38.0)	52 (76.5)	14 (24.1)	44 (38.9)	81 (40.3)	<0.001
Type 2 diabetes mellitus, n (%)	22 (19.6)	55 (25.1)	42 (21.9)	23 (33.8)	9 (15.5)	22 (19.5)	73 (36.3)	0.001
Dyslipidemia, n (%) ^‡^	48 (42.9)	108 (49.3)	64 (33.3)	33 (48.5)	16 (27.6)	37 (32.7)	79 (39.3)	0.003
Body mass index >30 kg/m^2^, n (%)	28 (25.0)	79 (36.1)	64 (33.3)	18 (26.5)	5 (8.6)	38 (33.6)	37 (18.4)	<0.001
Aspartate aminotransferase (IU/L), median (IQR)	26.0 (21.0, 36.0)	38.0 (29.0, 52.0)	49.5 (31.0, 70.5)	40.0 (28.0, 63.5)	22.0 (19.0, 27.0)	28.0 (22.0, 41.0)	21.0 (18.0, 24.0)	<0.001
Alanine aminotransferase (IU/L), median (IQR)	20.0 (13.0, 30.0)	27.0 (19.0, 50.0)	40.5 (23.0, 65.5)	24.0 (16.0, 36.0)	19.0 (13.0, 28.0)	45.0 (25.0, 60.0)	18.0 (13.0, 24.0)	<0.001
Prothrombin time (INR), median (IQR)	1.0 (1.0, 1.1)	1.1 (1.0, 1.1)	1.1 (1.0, 1.2)	1.1 (1.0, 1.2)	1.0 (0.9, 1.1)	1.1 (1.0, 1.2)	0.9 (0.9, 1.0)	<0.001
D-dimer (ng/mL), median (IQR)	297.0 (155.0, 536.0)	265.0 (171.0, 432.0)	391.0 (225.0, 760.0)	543.0 (216.0, 1653.0)	109.0 (50.0, 151.0)	719.0 (307.0, 1567.0)	ND ^§^	<0.001

* *p*-values were calculated using: Chi-squared test (male sex, chronic kidney disease, blood pressure, type 2 diabetes mellitus, dyslipidemia, and body mass index), one-way ANOVA (age and mean arterial pressure), and Kruskal–Wallis comparison (aspartate aminotransferase, alanine aminotransferase, prothrombin time, and D-dimer). ^†^ Defined as glomerular filtration rate <60 mL/min/m^2^. ^‡^ Defined during routine clinical care, supported by lipid biochemical measurement (cholesterol and triglycerides).^§^ Only one result was available, therefore median and interquartile range could not be calculated. ANOVA: analysis of variance; INR: international normalized ratio; IQR: interquartile range; IU: international units; ND: no data; chain reaction; SD: standard deviation.

**Table 3:** Overview of differences in biomarker levels between patients with SARS-CoV-2 infection (Groups 1–4) and ‘SARS-CoV-2 unexposed’ patients (Group 7).

Biomarker	Number of Samples			Biomarker Level (log_2_), Median (IQR)			Median Difference	*p*-Value	AUC (95% CI)
	SARS-CoV-2	‘SARS-CoV-2 Unexposed’ Patients	Total	SARS- CoV-2	‘SARS-CoV-2 Unexposed’ Patients	Total			
CRP	517	20	537	3.26 (1.85, 4.14)	−2.08 (−3.84, −1.51)	3.19 (1.55, 4.13)	5.341	<0.001	0.964 (0.948, 0.980)
GDF-15	500	201	701	11.39 (10.78, 12.25)	9.90 (9.24, 10.70)	11.07 (10.17, 11.94)	1.492	<0.001	0.830 (0.797, 0.863)
IL-6	567	201	768	5.56 (4.51, 6.64)	1.21 (0.58, 2.06)	4.90 (2.10, 6.22)	4.355	<0.001	0.949 (0.933, 0.964)
sACE2	591	201	792	−4.06 (−5.06, −2.00)	−2.84 (−5.06, −0.40)	−3.64 (−5.06, −1.69)	−1.222	<0.001	0.585 (0.539, 0.632)
sFlt-1	500	201	701	6.88 (6.56, 7.18)	6.45 (6.31, 6.58)	6.69 (6.44, 7.05)	0.431	<0.001	0.797 (0.764, 0.829)

AUC: area under the curve; CI: confidence interval; CRP: C-reactive protein; GDF-15: growth/differentiation factor-15; IL-6: interleukin-6; sACE2: soluble angiotensin-converting enzyme 2; sFlt-1: soluble fms-like tyrosine kinase-1.

**Table 4:** Overview of differences in biomarker levels between patients with SARS-CoV-2 infection who were admitted to hospital (Groups 2–4) and patients with SARS-CoV-2 infection who were discharged (Group 1).

Biomarker	Number of Samples			Biomarker Level (log_2_), Median (IQR)			Median Difference	*p*-Value	AUC (95% CI)
	Admitted	Discharged	Total	Admitted	Discharged	Total			
CRP	444	73	517	3.46 (2.21, 4.23)	1.33 (−1.15, 3.03)	3.26 (1.85, 4.14)	2.131	<0.001	0.775 (0.718, 0.832)
GDF-15	410	90	500	11.45 (10.90, 12.30)	10.97 (9.76, 12.02)	11.39 (10.78, 12.25)	0.485	<0.001	0.625 (0.551, 0.699)
IL-6	471	96	567	5.85 (4.93, 6.78)	3.79 (2.24, 5.12)	5.56 (4.51, 6.64)	2.059	<0.001	0.800 (0.750, 0.851)
sACE2	479	112	591	−4.64 (−5.06, −2.32)	−2.64 (−4.73, −0.91)	−4.06 (−5.06, −2.00)	−2.000	<0.001	0.648 (0.592, 0.704)
sFlt-1	410	90	500	6.96 (6.66, 7.24)	6.50 (6.23, 6.78)	6.88 (6.56, 7.18)	0.454	<0.001	0.751 (0.689, 0.813)

**Table 5:** Overview of differences in biomarker levels between patients with SARS-CoV-2 infection who were admitted to the ICU or died (Groups 3 and 4) and patients with SARS-CoV-2 infection who were admitted to the ward or discharged (Groups 1 and 2).

Biomarker	Number of Samples			Biomarker Level (log_2_), Median (IQR)			Median Difference	*p*-Value	AUC (95% CI)
	ICU or Died	Ward or Discharged	Total	ICU or Died	Ward or Discharged	Total			
CRP	249	268	517	3.74 (2.70, 4.42)	2.77 (1.32, 3.71)	3.26 (1.85, 4.14)	0.969	<0.001	0.670 (0.623, 0.716)
GDF-15	207	293	500	11.74 (11.09, 12.46)	11.15 (10.54, 11.94)	11.39 (10.78, 12.25)	0.593	<0.001	0.650 (0.602, 0.698)
IL-6	258	309	567	6.19 (5.21, 7.10)	5.12 (3.85, 6.05)	5.56 (4.51, 6.64)	1.073	<0.001	0.715 (0.673, 0.757)
sACE2	260	331	591	−4.64 (−5.06, −2.40)	−3.64 (−5.06, −1.71)	−4.06 (−5.06, −2.00)	−1.000	0.015	0.556 (0.511, 0.600)
sFlt-1	207	293	500	7.02 (6.71, 7.38)	6.77 (6.49, 7.05)	6.88 (6.56, 7.18)	0.252	<0.001	0.672 (0.624, 0.720)

## Data Availability

The study was conducted in accordance with applicable regulations. Ethical approval was provided by the Research Ethics Committee for Drug Research of the Vall d’Hebron University Hospital (Barcelona, Spain). An exemption from obtaining patient informed consent was granted by the Research Ethics Committee due to the health emergency presented by the COVID-19 pandemic. Therefore, participants of this study did not give consent for their data to be shared with additional third parties. For more information on the study and data sharing, qualified researchers may contact the corresponding author Francisco Rodriguez-Frias (frarodri@gmail.com).
